# Hot News Recommendation System from Heterogeneous Websites Based on Bayesian Model

**DOI:** 10.1155/2014/734351

**Published:** 2014-06-26

**Authors:** Zhengyou Xia, Shengwu Xu, Ningzhong Liu, Zhengkang Zhao

**Affiliations:** Department of Computer Science and Technology, Nanjing University of Aeronautics and Astronautics, China

## Abstract

The most current news recommendations are suitable for news which comes from a single news website, not for news from different heterogeneous news websites. Previous researches about news recommender systems based on different strategies have been proposed to provide news personalization services for online news readers. However, little research work has been reported on utilizing hundreds of heterogeneous news websites to provide top hot news services for group customers (e.g., government staffs). In this paper, we propose a hot news recommendation model based on Bayesian model, which is from hundreds of different news websites. In the model, we determine whether the news is hot news by calculating the joint probability of the news. We evaluate and compare our proposed recommendation model with the results of human experts on the real data sets. Experimental results demonstrate the reliability and effectiveness of our method. We also implement this model in hot news recommendation system of Hangzhou city government in year 2013, which achieves very good results.

## 1. Introduction

Owing largely to the ever-increasing volume and sophistication of information on the web, we are able to access an enormous amount of information from around the globe [1]. Online news reading has become a popular way to read news articles from a huge collection of news sources around the globe. Recommending news stories in personalized web services has become an active research direction with the development of Internet technologies for fast accessing real-time information around the world [2, 3]. Recommender systems are usually classified into three categories, based on how the recommendations are made [4, 5]: content-based recommendation, collaborative filtering, and hybrid. Content-based recommender systems: these recommender systems recommend an item to the user similar to the ones the user preferred in the past [6, 7]. Collaborative recommender systems: these systems recommend an item to the user based on the people with similar tastes and preferences who have liked it in the past. They have the advantage that they can recommend items for which little or no semantic information is available (music, movies, and products) [8–11]. Hybrid recommender systems: these systems combine both the collaborative and content-based recommendation techniques in order to improve the accuracy of the recommendation [12–14].

In this work, we study how to dynamically recommend hot news for group customers (e.g., government staffs) from hundreds of different heterogeneous news websites. Although Liu et al. [15] discuss recommending quality book reviews from heterogeneous websites, most previous studies about recommender systems based on person are presented in many other domains [1, 15–18]; little work is studied on hot news recommendations for group customers (government staffs). Generally, news recommender systems are based on a single website or are used to recommend news to a single user. The basic idea of current news recommender system is based on feedback or user's personal interests. For example, most websites recommend news to users based on article clicks, user's personal interests, and social network relation, which is called personalized recommendation. However, for group customers (government staffs), they are only interested in the top 20 (or 50) hottest pieces of news from the whole different websites without considering personal preferences. Therefore, previous personalized recommendation methods are not reliable in this scenario. The detailed reasons are as follows: (1) in China, since many news websites do not need the user to log in, user feedback, social network relation, and personal historical consumptions cannot be achieved; (2) clicks number on news of different websites cannot compare with each other, because there is no display or calculated clicks number on news in some Chinese news websites. Inspired by the above mentioned reasons, we proposed a real time hot news recommendation model based on text summarization and Bayesian model from different websites. In our model, we calculate the probability of hot news for every summary of news contents according to the joint probability equation and rank the probability results in descending order. We select the top 20 (or top 50) hottest news headlines from hundreds of news websites and recommend them to government staffs.

This paper is organized as follows: in Section 2, we present our hot news recommender system based on Bayes model in detail, including the motivation, algorithm. In Section 3, we first analyze our model on the real data set and prove the rationality of parameter selection in our model. Finally we give our conclusion in Section 3.

## 2. Hot News Recommendation Model Based on Bayesian Model

In the previous researches, we know that most news recommendations depend on clicks ranking or personal interests (or social relation). Click numbers on different news pages are effective only within the same website. We cannot compare hot degree of news from heterogeneous websites by click number of news. For many news websites, we can read online news without logging in. Since some problems exist in the current hot news recommendation from heterogeneous websites, we propose a model based on Bayesian model to recommend top hot news.

### 2.1. Bayesian Model

Imagine that documents are drawn from a number of classes of documents which can be modeled as sets of words where the (independent) probability that the *i*th word of a given document occurs in a document from class *C* can be written as

(1) $$\begin{array}{c} p\left(w_{i}\;|\;C\right). \end{array}$$

For this treatment, we simplify things further by assuming that words are randomly distributed in the document—that is, words are not dependent on the length of the document, position within the document with relation to other words, or other document-contexts.

Then the probability that a given document *D* contains all of the words *w*_*i*_, given a class *C*, is

(2) $$\begin{array}{c} p\left(D\;|\;C\right)=\underset{i}{\prod }p\left(w_{i}\;|\;C\right). \end{array}$$

The question that we desire to answer is “what is the probability that a given document *D* belongs to a given class *C*?” In order to answer the question, we define *p*(*D*∣*C*) and *p*(*C*∣*D*) as follows.

**Definition 1 .**

Consider

(3) $$\begin{array}{c} p\left(D\;|\;C\right)=\frac{p\left(D\cap C\right)}{p\left(C\right)}. \end{array}$$

**Definition 2 .**

Consider

(4) $$\begin{array}{c} p\left(C\;|\;D\right)=\frac{p\left(D\cap C\right)}{p\left(D\right)}. \end{array}$$

Bayes' theorem manipulates these into a statement of probability in terms of likelihood. Consider

(5) $$\begin{array}{c} p\left(C\;|\;D\right)=\frac{p\left(C\right)}{p\left(D\right)}p\left(D\;|\;C\right). \end{array}$$

Assume for the moment that there are only two mutually exclusive classes, *S* and ¬*S* (e.g., hot and not hot), such that every element (news) is in either one or the other:

(6) $$\begin{array}{c} p\left(D\;|\;S\right)=\underset{i}{\prod }p\left(w_{i}\;|\;S\right),\;\;p\left(D\;|\;\neg S\right)=\underset{i}{\prod }p\left(w_{i}\;|\;\neg S\right). \end{array}$$

Using the Bayesian result above, we can write

(7) $$\begin{array}{c} p\left(S\;|\;D\right)=\frac{p\left(S\right)}{p\left(D\right)}\underset{i}{\prod }p\left(w_{i}\;|\;S\right),\\ p\left(\neg S\;|\;D\right)=\frac{p\left(\neg S\right)}{p\left(D\right)}\underset{i}{\prod }p\left(w_{i}\;|\;\neg S\right). \end{array}$$

Dividing one by the other gives

(8) $$\begin{array}{c} \frac{p\left(S\;|\;D\right)}{p\left(\neg S\;|\;D\right)}=\frac{p\left(S\right)\prod_{i}p\left(w_{i}\;|\;S\right)}{p\left(\neg S\right)\prod_{i}p\left(w_{i}\;|\;\neg S\right)}=\frac{p\left(S\right)}{p\left(\neg S\right)}\underset{i}{\prod }\frac{p\left(w_{i}\;|\;S\right)}{p\left(w_{i}\;|\;\neg S\right)}. \end{array}$$

Thus, the probability ratio *p*(*S*∣*D*)/*p*(¬*S*∣*D*) can be expressed in terms of a series of likelihood ratios. The actual probability *p*(*S*∣*D*) can be easily computed from log⁡⁡(*p*(*S*∣*D*)/*p*(¬*S*∣*D*)) based on the observation that  *p*(*S*∣*D*) + *p*(¬*S*∣*D*) = 1.

Taking the logarithm of all these ratios, we have

(9) $$\begin{array}{c} \ln\frac{p\left(S\;|\;D\right)}{p\left(\neg S\;|\;D\right)}=\ln\frac{p\left(S\right)}{p\left(\neg S\right)}+\sum_{i}\ln\frac{p\left(w_{i}\;|\;S\right)}{p\left(w_{i}\;|\;\neg S\right)}. \end{array}$$

Finally, the document can be classified as follows. It is hot if *p*(*S*∣*D*) > *p*(¬*S*∣*D*) (i.e., ln⁡⁡(⁡*p*(*S*∣*D*)/*p*(¬*S*∣*D*)) > 0); otherwise, it is not hot.

### 2.2. Hot News Recommendation Algorithm Based on Bayesian Model

**Figure 1:**
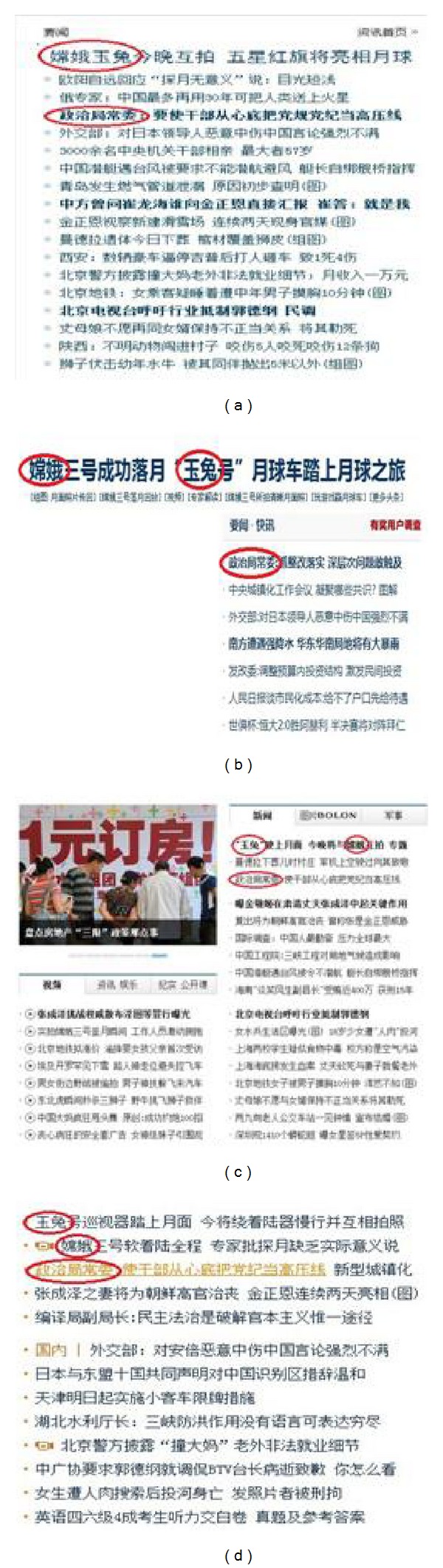
(a) News headline in http://www.ifeng.com/, (b) news headline in http://www.163.com/, (c) news headline in http://www.people.com.cn/, and (d) news headline in http://www.sina.com.cn/.

News headlines are generalization of news contents. Readers often can know the news content through this headline. This is because news headlines contain highly relevant keywords about news content. For news headline from different news websites, if the news contents are similar, they commonly exist in the same keywords as in headline, as shown in Figure 1.

As we can see in Figure 1, the vocabularies marked with red line are keywords in the current page. In four different websites, keywords of hot news are usually the same. Therefore, we firstly preprocess news headline. In this paper, we use the word segmentation technology to preprocess news headline. Each headline will be divided into vocabularies with nonfixed length and we calculate the statistic of the frequency of occurrence for each vocabulary. We define the frequency of occurrence of each vocabulary as vocabulary weight *w*, which is defined as follows.

**Definition 3 .**

Weight *w*: the frequency of occurrence of each vocabulary in all news headlines.

For different vocabulary, its probability that appears in the hot news is different. As shown in Figure 1, the vocabulary with high weight has high probability to appear in hot news. Therefore, we define an equation to calculate probability of vocabulary, which is shown as follows:

(10) $$\begin{array}{c} p\left(i\right)=0.9*\frac{w_{i}}{w_{\max}}, \end{array}$$

where *p*(*i*) is probability of vocabulary *i*. The value of *p*(*i*) shows hot degree of vocabulary. *i* represents the vocabulary *i*. *w*_*i*_ represents the weight of vocabulary *i*, *w*_max⁡_ represents the max weight among all vocabularies. 0.9 is coefficient of (10), which is gotten by experience.

For each message, we use the model to determine whether or not it is hot news. We use *E*_1_ to represent hot news event and *E*_2_ to represent not hot news event, which is shown in Table 1. *W*_1_ and *W*_2_ represent two vocabularies segmented from news headline with high weight. To facilitate discussion, we use two vocabularies *W*_1_ and *W*_2_ to describe the basic idea of our model based on Bayesian.

According to previous discussion, the vocabulary with high weight has high probability to appear in hot news. In Table 1, since *W*_1_ and *W*_2_ vocabularies with high weight exist in event *E*_1_, we consider that event *E*_1_ may be a hot news. Similarly, since *W*_1_ and *W*_2_ vocabularies with high weight do not exist in event *E*_2_, we consider that it may not be a hot news. In order to more accurately express our ideas of model, we use probabilistic methods to reexpress Table 1 as Table 2.

In Table 2, probability of *W*_1_ vocabulary with high weight existing in hot news is presented as *P*(*S*∣*W*_1_); probability of *W*_2_ vocabulary with high weight existing in hot news is presented as *P*(*S*∣*W*_2_). *P*(*S*) represents the probability that the news is a hot news. According to above description in Table 2, we can calculate the probability of event *E*_1_ and *E*_2_ as hot news by solving the joint probability. The calculation equations are shown as follows:

(11) $$\begin{array}{c} P\left(E_{1}\right)=P\left(SW_{1}\right)P\left(SW_{2}\right)P\left(S\right),\\ P\left(E_{2}\right)=\left(1-P\left(S\;|\;W_{1}\right)\right)\left(1-P\left(S\;|\;W_{2}\right)\right)\left(1-P\left(S\right)\right), \end{array}$$

where *P*(*E*_1_) represents the probability of *E*_1_ as a hot news and *P*(*E*_2_) represents the probability of *E*_2_ as a hot news. In the case of *W*_1_ vocabulary and *W*_2_ vocabulary, we get the probability of the hot news as follows:

(12) P=P(E1)P(E1)+P(E2)=(P(S ∣ W1)P(S ∣ W2)P(S))  ×(P(S ∣ W1)P(S ∣ W2)P(S)+(1−P(S ∣ W1))    ×(1−P(S ∣ W2))(1−P(S)))−1.

Let *P*(*S*) = 0.5, for (4), then we get the final equation:

(13) $$\begin{array}{c} P=\frac{P_{1}P_{2}}{P_{1}P_{2}+\left(1-P_{1}\right)\left(1-P_{2}\right)}, \end{array}$$

where *P*_1_ represents *P*(*S*∣*W*_1_) and *P*_2_ represents *P*(*S*∣*W*_2_).

We cannot determine whether the news is hot news only by using two vocabularies to analyze news headline in practical applications. Therefore, we should select appropriate number of vocabularies to analyze the news headline. Because length of every news headline is different, according to our practical experience data, we select four vocabularies with higher weight as keywords for a news headline. (We will discuss the reason of selecting four vocabularies in the next section.) For some news headline, their lengths are relatively short and could not be divided into four vocabularies. In this case, we add the default vocabularies to these news headlines. The probability of default vocabularies that exist in hot news is set to 0.4, which means that the default vocabularies are not important and are less than 0.5. The 0.4 is set by experience. Calculation equation of hot news probability finally is shown as follows:

(14) $$\begin{array}{c} P=\frac{P_{1}P_{2}P_{3}P_{4}}{P_{1}P_{2}P_{3}P_{4}+\left(1-P_{1}\right)\left(1-P_{2}\right)\left(1-P_{3}\right)\left(1-P_{4}\right)}, \end{array}$$

where probability of four vocabularies is, respectively, *P*_1_ *P*_2_ *P*_3_ *P*_4_, and *P* represents the probability of hot news. According to above discussion, the detailed algorithm of hot news recommendation is shown as Algorithm 1.

### 2.3. Experimental Results and Analysis

**Figure 2:**
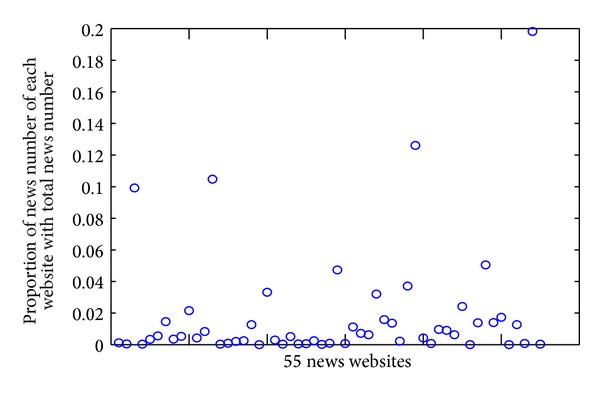
Distribution of news number for 55 different news websites.

In order to verify the reliability of our model, we do experiments on real data sets. We collected news from 55 different news websites, from 2013.10 to 2013.12, which includes 15243 pieces of news. Because there are different updating frequencies from news website, news from different websites has different proportions of the total number of news as shown in Figure 2.

**Figure 3:**
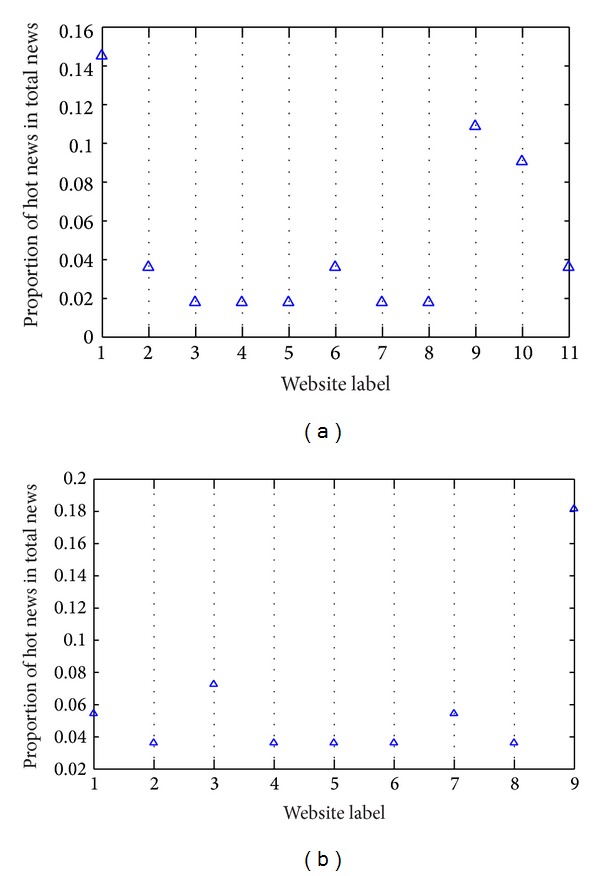
Proportion of news from different news websites ((a) December 1; (b) December 2).

In Figure 2, only a few websites publish more news. The vast majority of websites publish little news and the proportion of news number from these websites is below 0.06. This means that the number of news published and updated is less in most websites. We selected data of two days from our data set to further illustrate our above discussion, which is shown as in Figures 3(a) and 3(b).

**Figure 4:**
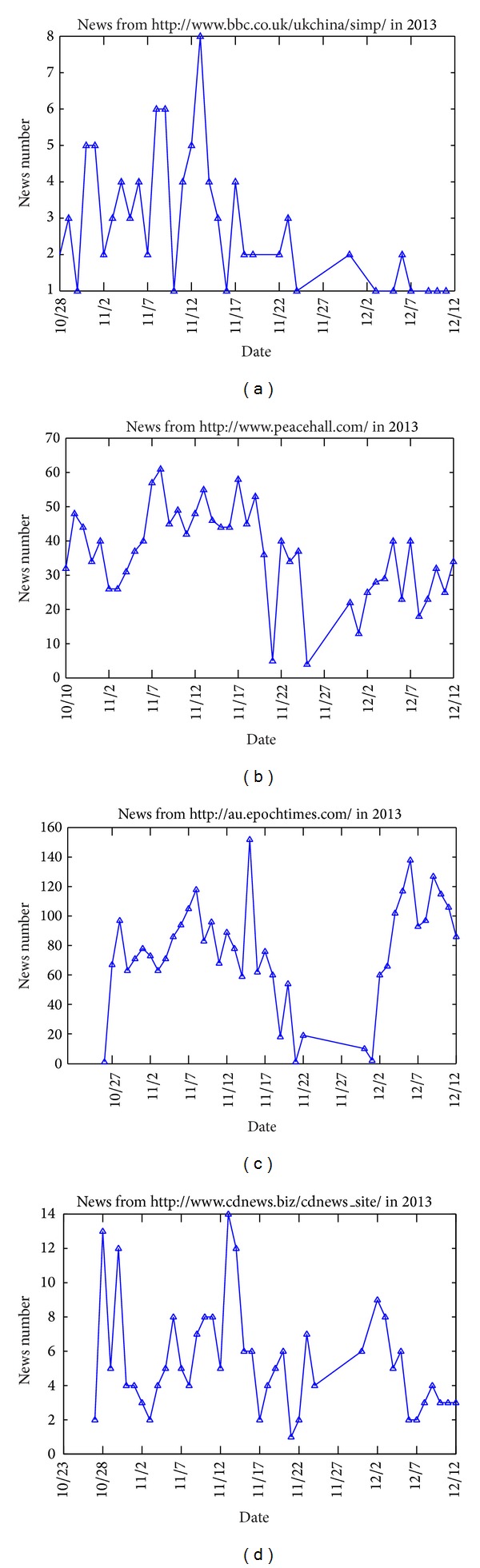
Relationship between news number and time for 4 different websites.

In Figure 3(a), we selected the data set on December 1 and 2, 2013. The 11 news websites updated news contents, and 8 of them are below 0.06.  Figure 3(b) (December 2) also reflects the situation. This means that not all websites publish a lot of news every day, and only a few number of news websites publish more news. In order to observe the relationship between news number and time for different websites, we selected four news websites from the data set, as shown in Figures 4(a), 4(b), 4(c), and 4(d).

In Figure 4, from October 2013 to December 2013, two websites (a) and (d) have relatively few number of news every day, and two websites (b) and (c) have more number of news every day. For each website, news number has great changes over time, but fluctuated curves of four news websites are similar. It shows that regularities of the four websites published news are similar.

**Figure 5:**
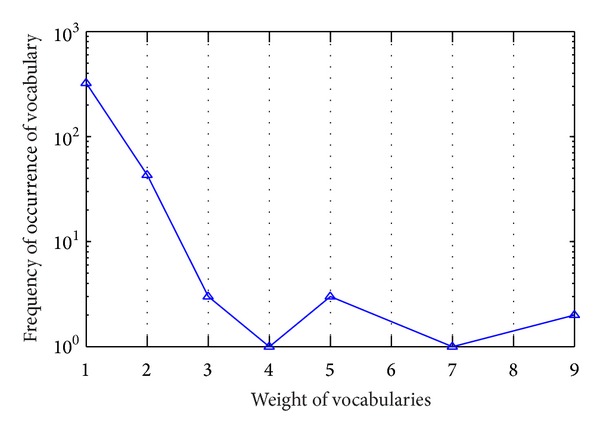
Relationship between weight of vocabularies and frequencies of weight of vocabularies.

The weight of vocabularies reflects frequency of vocabularies in news headline. According to (2) in the above section, weight of vocabularies is proportional to hot degree of news. Therefore, the weight of vocabulary is one of the important parameters. In Figure 5, we observed the relationships between weight of vocabularies and frequency of weight of vocabularies in one day.

**Figure 6:**
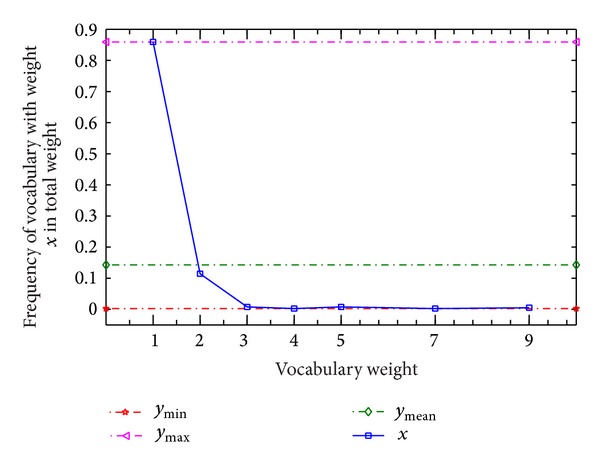
Relationship between vocabulary weight and frequency of vocabulary.

In Figure 5, vocabularies with high weight have less frequency appearing in news headline, such as frequencies of vocabularies with weight value of 3 to 9 are less than 10. Frequencies of vocabularies with weight value of 1 are greater than 100. This phenomenon is consistent with the actual situation. In reality, news headline from different news websites may contain same vocabularies, and weights of these vocabularies will be relatively larger. News from different news websites has different focus; therefore, quantity of similar news is in the minority. Generally, these minority pieces of news are regarded as the hot news; namely, hot degree of news is related with weight of vocabularies.  Figure 6 further verifies our ideas.

In Figure 6, the maximum of *y* axis and the minimum of *y* axis have apparent difference. In Figure 6, we can obviously observe that the number of vocabularies with weight of 1 makes up the vast majority in total vocabularies, which is close to 0.9. The number of vocabularies with high weight is almost equal to zero.  Figure 6 demonstrates our idea.

**Figure 7:**
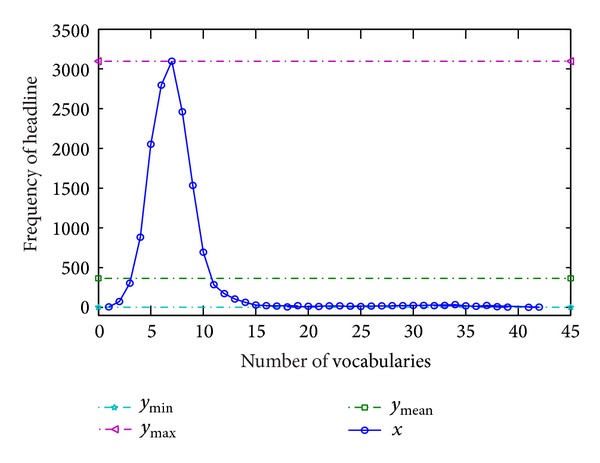
Relationship between number of vocabularies and frequency of news headline.

In (14) of the above section, for each news headline, we selected four vocabularies with most high weight to calculate the probability.  Figure 7 gives the reason of why we choose four vocabularies as the threshold of our Bayesian model.

In Figure 7, when news headlines are divided into vocabularies, very few numbers of news headlines consist of vocabularies less than 4. The vast majority of news headlines can split out the four vocabularies or more.  Figure 7 shows that about three hundred pieces of news cannot be split out into four vocabularies in total of 15243 pieces of news. If we use the five vocabularies, the two thousand pieces of news cannot be split out. Therefore, using five vocabularies and more in our model is not suitable because several thousand pieces of news cannot be split out. In actual situation, news headlines with less than four vocabularies are often difficult to summarize the news content. Too many vocabularies often make the headline too complex. Therefore, we determine whether the news is hot news through calculating the joint probability of four vocabularies.

**Figure 8:**
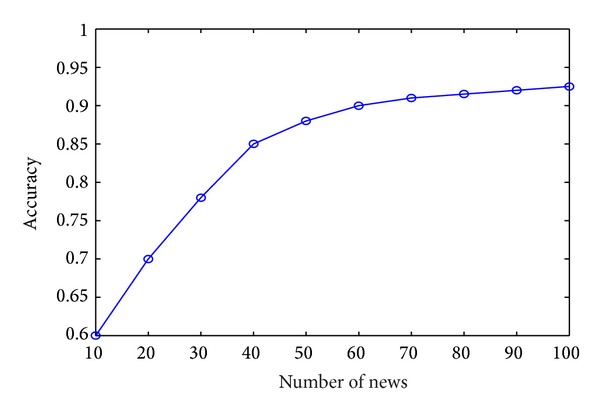
Similarity results of our model and human experts.

In order to verify the validity and reliability of our model, we randomly choose 2000 pieces of news from our news data set. Since there is little research work about hot news recommendation from plenty of different news websites, we cannot find related algorithms about our research. Therefore, we cannot compare our model with other previous works. We have to compare our model with human expert's selection. We invited five news staffs to select top 10, 20, 30, 40, 50, 60, 70, 80, 90, and 100 hot pieces of news from the 2000 news data sets. We compare similarity of these two result sets, as shown in Figure 8.

In Figure 8, when we select the hottest top 10 pieces of news, similarity of the result of our method and human experts is relatively low because when the news quantity is less, the human experts' subjective influences will make a difference for hot news. And with the increase of number of news, human experts' subjective influences will decrease accordingly. Therefore, when the number of hot news reaches a certain value, the result of our model and human experts is similar. For these 2000 pieces of news, when the top number of hot news is 100, precision of results is ideal.

## 3. Conclusion

In this paper, we investigate the problem of hot news recommendation from plenty of heterogeneous news websites and try to resolve some critical issues of this problem, for example, without click number, personal history, and personal interests. To do so, we explore the hot news recommendation from different news websites based on Bayesian model. We do plenty of experiments on real data sets to illustrate the basic idea and motivation of our model. We compare the result of our model with the result of human news staffs. The experimental results show that our model can effectively extract top hot news from news data sets which are collected from different websites. We implement our model in hot news recommendation system of Hangzhou city government in 2013. Our hot news recommendation system dynamic provides top 30 oversea pieces of news, top 50 domestic pieces of news, and top 30 local city pieces of news from Hangzhou city government. Currently, our model cannot deal with “black hole phenomenon,” which means that news content is basically the same, but they have completely different headlines. For future work, we plan to investigate the “black hole phenomenon” of our model and propose the better methods to solve it.

## Figures and Tables

**Algorithm 1:**
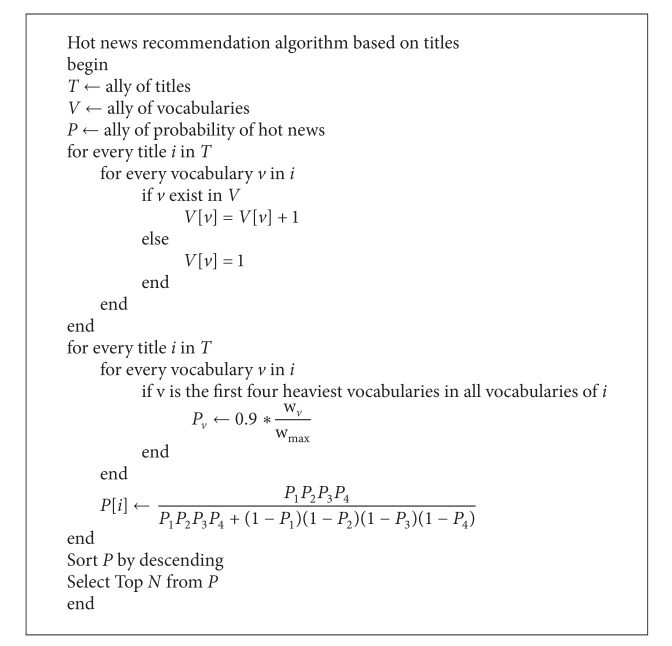
Hot news recommendation algorithm.

**Table 1:** High weight vocabulary and hot news.

Event	*W*_1_	*W*_2_	Hot news
E_1_	Exist	Exist	Yes
E_2_	Not exist	Not exist	No

**Table 2:** Vocabulary with high weight existing probability and hot news probability.

Event	W_1_	W_2_	Hot news
E_1_	P(S∣W_1_)	P(S∣W_2_)	P(S)
E_2_	1 − P(S∣W_1_)	1 − P(S∣W_2_)	1 − P(S)
